# Construct validation of patient global impression of severity (PGI-S) and improvement (PGI-I) questionnaires in the treatment of men with lower urinary tract symptoms secondary to benign prostatic hyperplasia

**DOI:** 10.1186/1471-2490-12-30

**Published:** 2012-11-07

**Authors:** Lars Viktrup, Risa P Hayes, Ping Wang, Wei Shen

**Affiliations:** 1 Lilly Research Laboratories, Eli Lilly and Company, Indianapolis, IN 46285, USA

**Keywords:** Patient global impression scale, Lower urinary tract symptoms, Construct validity

## Abstract

**Background:**

Lower urinary tract symptoms (LUTS) in aging men are often associated with benign prostatic hyperplasia (BPH). While regulatory evaluations of treatment benefit require an assessment of specific symptoms, a simpler approach to measuring patients’ perceptions of severity and symptom change may be particularly useful for clinical practice. The aim of this study was to provide evidence of the validity of the 1-item Patient Global Impression of Severity (PGI-S) and Improvement (PGI-I) questionnaires for use as outcome measures in the treatment of BPH-LUTS.

**Methods:**

This was a secondary analysis of data from 4 randomized placebo-controlled 12-week trials evaluating tadalafil for the treatment of BPH-LUTS (N=1694). Visit 2 (V2 [beginning of a 4-week placebo lead-in period]) and endpoint assessments included International Prostate Symptom Score (IPSS), IPSS Quality of Life Index (IPSS-QoL), BPH Impact Index (BII), and peak urine flow (Qmax). PGI-S was only administered at V2 and PGI-I only at endpoint. Associations between the PGI-S or the PGI-I and the other assessments were analyzed by calculating Spearman rank correlation coefficients and performing analysis of variance (ANOVA).

**Results:**

Spearman correlation coefficients were 0.43, 0.43, 0.53, and −0.09, between the PGI-S and IPSS, IPSS-QoL, BII, and Qmax baseline results (all P<0.001). Similar results were seen across race, ethnicity, and baseline severity (moderate LUTS versus severe LUTS). IPSS, IPSS-QoL, BII baseline scores (P <0.001) and Qmax values (P=0.003) were significantly different among the 4 PGI-S severity levels. Spearman correlation coefficients were 0.56, 0.53, 0.47 and −0.15 between the PGI-I and change in IPSS, IPSS-QoL, BII scores, and Qmax values from baseline to endpoint (all P<0.001). Similar results were seen across race, ethnicity, and baseline severity. Change in IPSS, IPSS-QoL, BII scores, and Qmax values (P<0.001) were significantly different among the PGI-I levels (i.e., patient perception of change in urinary symptoms).

**Conclusions:**

This study demonstrated patients’ overall perceptions of their severity and change in BPH-LUTS can be captured in a way that is simple, valid, and easily administered in a research setting or clinical practice. Clinical parameters are weakly associated with patients’ perception of urinary symptoms, emphasizing the importance of a patient-reported assessment in the evaluation of BPH-LUTS treatment benefit.

## Background

Lower urinary tract symptoms (LUTS), often associated with benign prostatic hyperplasia (BPH), are common in aging men worldwide with a severity that seems to be similar across countries [1]. Symptoms may belong to 1 of 3 domains: voiding (obstructive) symptoms which include slow stream, splitting/spraying of stream, intermittency, hesitancy, straining, and terminal dribble; storage (irritative) symptoms which include frequency, nocturia, urgency, and incontinence; and postmicturition symptoms which include feeling of incomplete emptying and postmicturition dribble [2]).

Several validated questionnaires have been developed in collaboration with The American Urology Association (AUA) to assess the severity and treatment effect in men with BPH-LUTS. One such instrument is the International Prostate Symptom Score (IPSS) which is commonly used to assess therapeutic efficacy of BPH therapy [3,4] and has been translated into multiple languages. The IPSS QoL Index is an additional question usually included at the end of the IPSS questionnaire, though not part of the total IPSS score [5]. The AUA committee also developed the BPH Impact Index (BII) to assess the impact of LUTS suggestive of BPH on patient health and functioning [4]. The BII has recently been further validated based on several tadalafil clinical studies [6,7]. While the IPSS questionnaire is accepted as a critical component in BPH-LUTS research, the IPSS-QoL and BII (assessments of symptom burden and impact) are less frequently used.

In light of the need for a simple and easy-to-use validated questionnaire to assess patients’ overall perception of their condition, the Patient’s Global Impressions of Severity (PGI-S) and Patient’s Global Impressions of Improvement (PGI-I) questionnaires were included in several tadalafil BPH-LUTS clinical studies conducted worldwide. The PGI-S and PGI-I respectively are 1-item questionnaires that ask an individual patient to rate the severity of a specific condition (single-state scales) at baseline and or to rate at endpoint the perceived change in his/her condition in response to therapy (transition scales) [8,9]. These types of measures have been implemented and/or validated in clinical studies of patients with stress incontinence [9], urogenital prolapse [10] and other non-urological diseases [11]. The aim of this study was to provide evidence that supports the validity of these two 1-item questionnaires for LUTS suggestive of BPH by correlating them with other subjective and objective measures of symptom severity or changes in severity in tadalafil placebo-controlled studies.

## Methods

### Study design and participants

This was a secondary analysis of data from 4 clinical trials. Details of the study designs and populations have previously been published [12-15].

Study 1 (LVHJ) was a randomized, double-blind, placebo-controlled, parallel-design multinational study to evaluate the efficacy and safety of daily tadalafil 5 mg once daily for 12 weeks. Study participants were men with LUTS secondary to BPH (BPH-LUTS) residing in Argentina, Germany, Italy, Mexico, or the United States (Clinicaltrials.gov: NCT00827242 [12]).

Study 2 (LVHR) was a randomized, double-blind, 3-group, placebo-controlled, parallel-design, multinational study to evaluate the efficacy and safety of tadalafil 2.5- and 5-mg once-daily dosing for 12 weeks for the treatment of erectile dysfunction (ED) and BPH-LUTS. Study participants were men with both ED and BPH-LUTS residing in Canada, France, Germany, Greece, Italy, Mexico, Portugal, Russia, and the United States (Clinicaltrials.gov: NCT00855582 [13]).

Study 3 (LVHB) was a randomized, double-blind, 4-group, placebo and tamsulosin 0.2 mg controlled, parallel design, multinational study to evaluate the efficacy and safety of tadalafil 2.5 mg and 5 mg once-a-day dosing for 12 weeks in men with BPH-LUTS residing in Japan, Korea, and Taiwan (Clinicaltrials.gov: NCT00861757 [14]).

Study 4 (LVHT) was a randomized, double-blind, 3-group, placebo-controlled, parallel-design, pilot study to evaluate the efficacy and safety of tadalafil 5 mg and tamsulosin 0.2 mg once-a-day dosing for 12 weeks in men with BPH-LUTS residing in Korea (Clinicaltrials.gov: NCT00540124 [15]).

Men who were at least 45 years of age, with moderate to severe LUTS due to BPH and evidence of bladder obstruction, were eligible to participate in all 4 studies. ED was an entry criterion only in Study 2 (LVHR). The 4 studies were in compliance with the Helsinki Declaration.

In each study, participants were screened at V1. In all 4 studies, there was a 2- (Study 3, LVHB) or 4-week screening/washout period (V1-V2), a 4-week placebo lead-in period (V2-V3), and a 12-week double-blinded treatment period (V3 to last visit). At V2, participants in all studies were required to have an IPSS ≥13 and a uroflowmetry measure of urinary peak flow rate (Qmax) ≥4 to ≤15 ml/second on a voided volume of 125 mL to continue in the study. One study (Study 3, LVHB) also required a minimum total prostate volume of 20 mL. V3 (randomization) initiated the double-blind active-treatment placebo-controlled 12-week period. Patient characteristics were assessed at V2.

#### Clinical measures

Postvoid residual volume (PVR) and prostate-specific antigen (PSA) were assessed in all studies, but not consistently carried out at the V2. The change in peak urine flow rate (Qmax) from randomization (V3) to endpoint was a secondary objective in all the studies. Changes in Qmax included in this validation were based on assessment at V2 and at endpoint.

#### Patient-reported measures

The change in IPSS from randomization (V3) to endpoint was the primary objective in all the studies; changes in IPSS-QoL, BII and Qmax from V3 to endpoint were secondary objectives. Changes in these patient-reported outcomes included in this validation were based on assessment at V2 and at endpoint.

The PGI-I also was a secondary objective, with 1 assessment at endpoint. PGI-S assessed participants’ perception of symptom severity at V2 and was an exploratory parameter. The Korean pilot study (Study 4) did not assess BII at V2 and the BII scores could therefore not be used from this study to correlate with PGI-S.

The IPSS is a self-administered 7-item urinary symptom severity scale about symptoms occurring over the past month (Additional file 1; International Prostate Symptom Score). The urinary symptoms can be categorized into voiding, storage and post-micturition symptoms. Item scores are summed for a total IPSS score that ranges from 0 to 35 with a higher score indicating more severe symptoms.

The IPSS-QoL is a single question: “If you were to spend the rest of your life with your urinary condition just the way it is now, how would you feel about that?” with responses scored as follows: “delighted” (0), “pleased” (1), “mostly satisfied” (2), “mixed about equally satisfied and dissatisfied” (3), “mostly dissatisfied” (4), “unhappy” (5), and “terrible” (6) (Additional file 1; International Prostate Symptom Score).

The BII is a self-administered validated questionnaire with 4 questions about urinary problems during the past month regarding physical discomfort, worry about health, how bothersome symptoms are, and whether the symptoms are interfering with doing usual activities (Additional file 2; BPH Impact Index). The first 3 questions are scored from 0 to 3, while the fourth is scored from 0 to 4. Item scores are summed for a BII total score that ranges from 0 to 13, with higher scores indicating more bother or problems associated with urinary symptoms within the previous month.

PGI-I is a 1-item questionnaire designed to assess the patient’s impression of changes in urinary symptoms. The PGI-I asks the patient to best describe how his urinary symptoms are now, compared with how they were before he began taking medication in the study. (“*Check the one number that****best describes how your urinary symptoms are now, compared with how they were before you began taking medication in this study***”). The patient enters his answer on a 7-point scale scored as: (1) “very much better,” (2) “much better,” (3) “a little better,” (4) “no change,” (5) “a little worse,” (6) “much worse,” or (7) “very much worse.” Examples of urinary symptoms were provided based on the symptoms in the IPSS questionnaire [3] with the addition of terminal dribble and accidental urinary leakage [16]: “Urinary symptoms include difficulties in postponing urination, having to push or strain to begin urination, a weak urinary stream, stopping and restarting urination several times when attempting to urinate, prolonged urination with the end of urine flow slowing to a trickle (terminal dribble), the feeling that you haven’t emptied your bladder after you have finished urinating, having to urinate again less than 2 hours after the last time you finished urinating, accidental urinary leakage, or having to get up at night to urinate”. The PGI-I was administered once at the end-of-study visit (Week 12 or the last visit).

PGI-S is a 1-item questionnaire designed to assess patient’s impression of disease severity. The PGI-S item asks the respondent to best describe how his urinary symptoms are now (“*Check the one number that****best describes how your urinary symptoms are now***”) on a 4-point scale scored as: “normal” (1), “mild” (2), “moderate” (3), or “severe” (4) . Examples of urinary symptoms similar to the PGI-I were provided. The PGI-S was administered once at the beginning of the placebo lead-in period (V2).

All patient-reported measures were originally developed in English for the United States. Therefore, linguistic validations that included forward and backward translations, review/reconciliation, and cognitive debriefing with targeted patients were conducted for primary languages spoken in the countries participating in the 4 trials. In addition, for most linguistic validations, harmonization, a meeting in which all translations and the original are compared to ensure cross-cultural equivalence of concepts and the use of colloquial language, was performed.

### Statistical analysis

The study results for tadalafil efficacy versus placebo are reported elsewhere [12-15].

In the current paper, because the aim of the analysis was not to make conclusions about tadalafil efficacy, but rather to establish construct validity of the PGI-S and the PGI-I, tadalafil and placebo data from the 4 clinical studies were pooled (N=1694) and analyzed. Furthermore, an evaluation of tadalafil efficacy versus placebo would be based on changes from randomization (V3) to endpoint, while the validation of the PGI-I per its question ( see above) was based on changes from treatment onset at V2 (initiating with single-blind placebo therapy) to endpoint.

For a single-item global assessment to adequately (i.e., validly) assess a patient’s overall appraisal of their condition of change in their condition, its single score would be expected to capture not only symptom burden but also impact of that symptom burden on the patient’s life. Therefore, we hypothesized that the PGI-S and PGI-I scores would be significantly and meaningfully associated with the IPSS, IPSS-QoL, and BII scores. In addition, we explored the relationship with Qmax, the only clinical measure consistently assessed at V2 in the 4 clinical studies.

The associations between PGI-S response at V2 and other patient-reported and clinical measures at V2 (IPSS, IPSS-QoL, BII, and Qmax) were evaluated using 2 types of analyses. First, Spearman rank correlation coefficients were calculated between PGI-S and the other measures. Spearman correlation coefficient is a nonparametric analysis which assesses how well the relationship between 2 variables can be described using a monotonic function [17]; for example, whether patients’ perception of greater severity, as measured by the PGI-S, is associated with greater symptom burden as measured by IPSS. Correlation coefficients range from −1.0 to 1.0. Using a criterion suggested by Guilford and Fruchter [18], a significant correlation coefficient ≤−0.30 or ≥0.30 between the PGI-S and the V2 assessment of other patient-reported or clinical measures (i.e., supportive of the validity of the PGI-S).

The second type of analysis, unadjusted one-way analysis of variance (ANOVA), was performed to evaluate the differences of these measures among the 4 PGI-S categories and pair-wise comparisons were made. Identical ANOVA analyses, adjusted for covariates (e.g., age, prior alpha blocker use, country, baseline LUTs severity) were performed to confirm results. For all analyses, the a priori hypotheses were that patients perceiving more severe disease measured by PGI-S would have higher (worse) IPSS, IPSS-QoL, and BII scores and lower (worse) peak urine flow rate at V2.

The associations between PGI-I response at last visit and change in IPSS, IPSS-QoL, BII, and Qmax values from V2 to the last visit were evaluated using the same 2 types of analysis as performed with the PGI-S. For all analyses, the a priori hypotheses were that patients who reported change in their urinary symptoms would have better or worse IPSS, IPSS-QoL, and BII scores or Qmax values consistent with the direction of their perceived change in symptoms as measured by the PGI-I.

To further provide support for the validity of the PGI-S and PGI-I, the correlation analyses were repeated across 6 racial, ethnic, and severity subgroups: Asians versus Caucasians, Hispanic Caucasians versus non-Hispanic Caucasians, and LUTS severity at V2 (per IPSS classification of moderate versus severe). Assessment of race and ethnic subgroups was restricted within the relevant regions: Asians were only from Asian studies; Caucasians, including both Hispanic and non-Hispanic Caucasians, were only from non-Asian studies). It should be noted that these analyses were conducted to support the validity of the PGI-S and PGI-I and not to explore whether ethnic/racial groups differ in perception of severity or response to treatment. For all analyses, participants were included regardless of what type of treatment they received.

## Results

### Participant characteristics

A total of 1694 men were enrolled and subsequently randomized in the 4 studies; mean age was 63.1 years (SD = 8.2, range from 45 to 87). A total of 35.6% had severe symptoms of BPH-LUTS per IPSS and 48.6% had severe outlet obstruction per urinary peak flow rate (Qmax) (Table 1). Participant characteristics obtained at V2 were generally similar across studies; although in Study 2 (LVHR), more patients had severe outlet obstruction per Qmax, whereas in Study 4 (LVHT), more patients and their clinicians reported severe symptoms (data not shown).

**Table 1:** Baseline Characteristics and Demographics

**Baseline Characteristics**	**Statistics**
Total number of randomized patients, N	1694
Age, mean (SD)	63.1 (8.2)
Peak urine flow rate (Qmax)	
Mild (Qmax > 15 ml/s), n (%)	11 (0.7%)
Moderate (Qmax 10–15 ml/s), n (%)	837 (50.7%)
Severe (Qmax < 10 ml/s), n (%)	802 (48.6%)
PSA (mg/L), mean (SD)	1.8 (1.6)
PVR (mL), mean (SD)	38.1 (42.0)
LUTS Severity	
Moderate (IPSS 8–19), n (%)	1090 (64.4%)
Severe (IPSS >= 20), n (%)	603 (35.6%)
Race/Ethinity	
Asian, n (%)	763(45.0%)
Caucasian, n (%)	861(50.8%)
Hispanic Caucasian, n (%)	164 (9.7%)
Non-Hispanic Caucasian, n (%)	697 (41.1%)
Other Race, n (%)	70 (4.1%)

SD: standard deviation; n: number of patients; PSA: Prostate-specific antigen; PVR: post-void residual urine volume; LUTS: Lower Urinary Tract Symptoms; Asian: from the 2 studies conducted in Asia; Caucasian/Other race: from the 2 studies conducted outside Asia. Values are from V1-V2, before enrolled into the single-blind placebo run in period.

A total of 1692 men completed the PGI-S at V2 (before the placebo run-in period) and 1628 men completed the PGI-I at last visit. Participants’ self-reported ratings of urinary symptom severity using the PGI-S were 1.1% normal, 22.2% mildly abnormal, 61.4% moderately abnormal, and 15.3% severely abnormal. The mean symptom scores for all participants for IPSS, IPSS-QoL, BII (calculated using only data from Study 1, 2, and 3) at V2 were 19.8, 4.1 and 6.3, respectively. The mean V2 Qmax value was 10.0 ml/s.

### Construct validity of the PGI-S

Spearman correlation coefficients calculated between PGI-S and all variables were statistically significant (P<0.001) and in the direction hypothesized (Table 2). However, the correlation coefficient calculated between PGI-S and Qmax did not reach the criterion of ≤−0.30 designated a priori as supportive of validity (Table 2). Similar trends of correlations were observed across ethnicity, race and baseline severity (Table 3).

**Table 2:** Correlation between PGI-S and other baseline BPH Measures in all patients

**PGI-S (baseline visit)**	**n (%)**	**Mean IPSS (SD)**	**Mean IPSS-QoL (SD)**	**Mean BII (SD)**	**Mean Qmax (SD)**
Normal	18 (1.1%)	14.9 (2.9)	2.4 (0.6)	1.1 (1.4)	10.4 (2.4)
Mild	376 (22.2%)	16.8 (3.9)	3.5 (1.1)	4.0 (2.4)	10.4 (2.7)
Moderate	1039 (61.4%)	19.8 (4.7)	4.1 (1.0)	6.7 (2.5)	9.9 (2.8)
Severe	259 (15.3%)	24.2 (5.5)	5.0 (0.9)	9.1 (2.6)	9.6 (2.9)
Overall comparison p-value		<0.001	<0.001	<0.001	0.003
Pairwise comparison p-value					
Normal vs. Mild		0.092	<0.001	<0.001	0.993
Normal vs. Moderate		<0.001	<0.001	<0.001	0.461
Normal vs. Severe		<0.001	<0.001	<0.001	0.273
Mild vs. Moderate		<0.001	<0.001	<0.001	0.002
Mild vs. Severe		<0.001	<0.001	<0.001	<0.001
Moderate vs. Severe		<0.001	<0.001	<0.001	0.171
Spearman’s Correlation		0.43**	0.43**	0.53**	−0.09**

PGI-S: Patient Global Impression of Severity; IPSS: International Prostate Symptom Scores; IPSS QoL: IPSS-Quality of Life (QoL), BII - Benign Prostatic Hyperplasia Impact Index; Qmax: peak urine flow rate; n: number of patients; SD: Standard Deviation. Overall and pairwise comparison p-values are from analysis of variance without adjusting for covariates. **: p<0.001.

**Table 3:** Correlation between PGI-S and other baseline BPH Measures across race, ethnicity and baseline severity

**Spearman’s Correlation**	**n**	**IPSS**	**IPSS-QoL**	**BII**	**Qmax**
Asian	763	0.45**	0.45**	0.58**	−0.06
Caucasian	860	0.42**	0.43**	0.50**	−0.12**
Hispanic Caucasian	164	0.55**	0.54**	0.55**	−0.14
Non-Hispanic Caucasian	696	0.38**	0.38**	0.49**	−0.12*
Moderate LUTS	1088	0.35**	0.37**	0.49**	−0.04
Severe LUTS	603	0.36**	0.39**	0.50**	−0.11*

Asian: from the 2 studies conducted in Asia; Caucasian: from the 2 studies conducted outside Asia.

PGI-S: Patient Global Impression of Severity; IPSS: International Prostate Symptom Scores; IPSS QoL: IPSS-Quality of Life (QoL), BII - Benign Prostatic Hyperplasia Impact Index; Qmax: peak urine flow rate; LUTS: Lower Urinary Tract Symptoms; n: number of patients. **: p<0.001; *: p<0.05.

Overall comparisons among PGI-S severity responses showed significant differences across all variables regardless of whether the ANOVA model was unadjusted or adjusted for covariates. The mean IPSS score for the participants who responded “moderate” (19.8) or “severe” (24.2) on the PGI-S were significantly different (P<0.001) from the mean scores for all other responses with greater perceived severity corresponding to higher self-reported symptom burden. However, the mean IPSS scores for participants who responded “normal” (14.9) or “mild” (16.8) were significantly different from the mean scores for “moderate” and “severe” but not from each other. Mean IPSS-QoL and BII scores for the PGI-S categories were all significantly different from one another (P<0.001) with greater perceived severity corresponding to perception of worse quality of life and greater disease impact. Qmax values were not significantly different between participants who responded “normal” and those who responded “mild”, or “moderate”, or “severe” on the PGI-S, nor were they significantly different between the participants who responded “moderate” and those who responded “severe”. Only the Qmax values for those participants who responded “mild” were significantly different from the values for those who responded “moderate” or “severe”.

### Construct validity of the PGI-I

Spearman correlation coefficients calculated between PGI-I and all variables were statistically significant (P<0.001) and in the direction hypothesized (Table 4). However, as with the PGI-S, the correlation coefficient calculated between PGI-I and Qmax change did not reach the criterion of ≤−0.30 designated a priori as supportive of validity (Table 4). Similar trends of correlations were observed across ethnicity, race and V2 severity (Table 5).

**Table 4:** Correlation between PGI-I and other BPH Measures (change from baseline) in all patients

**PGI-I (last visit)**	**n (%)**	**Mean IPSS change (SD)**	**Mean IPSS-QoL change (SD)**	**Mean BII change (SD)**	**Mean Qmax change (SD)**
Very Much Better	99 (6.1%)	−14.4 (5.9)	−2.6 (1.6)	−5.2 (3.1)	3.7 (4.9)
Much Better	443 (27.2%)	−10.9 (5.6)	−2.0 (1.3)	−4.0 (2.9)	4.0 (5.6)
A Little Better	632 (38.8%)	−7.0 (5.4)	−1.0 (1.2)	−2.3 (2.7)	3.0 (5.0)
No Change	357 (21.9%)	−3.3 (5.2)	−0.3 (1.1)	−0.9 (2.7)	2.1 (4.3)
A Little Worse	65 (4.0%)	−0.2 (5.7)	0.0 (1.2)	−0.1 (3.0)	2.0 (4.8)
Much Worse	26 (1.6%)	3.5 (5.4)	0.5 (1.1)	1.8 (2.6)	1.3 (3.1)
Very Much Worse	6 (0.4%)	6.8 (5.1)	1.0 (0.9)	4.5 (2.1)	0.1 (2.0)
Overall comparison p-value		<0.001	<0.001	<0.001	<0.001
Pairwise comparison p-value					
Better vs. No change		<0.001	<0.001	<0.001	<0.001
Better vs. Worse		<0.001	<0.001	<0.001	0.002
No change vs. Worse		<0.001	<0.001	<0.001	0.534
Spearman’s Correlation		0.56**	0.53**	0.47**	−0.15**

PGI-I: Patient Global Impression of Improvement; IPSS: International Prostate Symptom Scores; IPSS QoL: IPSS-Quality of Life (QoL), BII - Benign Prostatic Hyperplasia Impact Index; Qmax: peak urine flow rate; n: number of patients; SD: Standard Deviation. Overall and pairwise comparison p-values are from analysis of variance without adjusting for covariates. **: p<0.001.

**Table 5:** Correlation between PGI-I and other BPH Measures (change from baseline) across race, ethnicity and baseline severity

**Spearman’s Correlation**	**n**	**IPSS**	**IPSS-QoL**	**BII**	**Qmax**
Asian	747	0.55**	0.52**	0.43**	−0.15**
Caucasian	816	0.57**	0.54**	0.49**	−0.13**
Hispanic Caucasian	158	0.65**	0.45**	0.49**	−0.15
Non-Hispanic Caucasian	658	0.55**	0.54**	0.49**	−0.14*
Moderate LUTS	1050	0.53**	0.52**	0.44**	−0.12**
Severe LUTS	577	0.62**	0.54**	0.51**	−0.19**

Asian: from the 2 studies conducted in Asia; Caucasian: from the 2 studies conducted outside Asia.

PGI-I: Patient Global Impression of Improvement; IPSS: International Prostate Symptom Scores; IPSS QoL: IPSS-Quality of Life (QoL), BII - Benign Prostatic Hyperplasia Impact Index; Qmax: peak urine flow rate; LUTS: Lower Urinary Tract Symptoms; n: number of patients. **: p<0.001; *: p<0.05.

Overall comparisons among PGI-I responses showed significant differences for all variables regardless of whether the ANOVA model was unadjusted or adjusted for the covariates. However, to conduct pairwise comparisons, the small numbers for some responses (e.g., “Very much worse” N=6) necessitated the collapsing of the 3 PGI-I response categories representing improvement (“a little better,” “much better,” and “very much better”) and the corresponding response categories representing worsening into 1 category each, “Better” and “Worse,” respectively. Mean IPSS, IPSS-QoL and BII change scores were significantly different (P<0.001) among all 3 response categories with improvement in symptoms, perceived QoL, and BPH impact corresponding to PGI-I “Better” responses, and worsening in symptoms, perceived QoL, and BPH impact corresponding to PGI-I “Worse” responses. Mean change in Qmax values for participants who responded on the PGI-I as improved or“Better” were significantly higher than those for participants who responded as “No Change” or classified as responding “Worse”. However, there were no significant differences between those who responded “No Change” and those who responded “Worse”.

## Discussion

In men participating in clinical trials evaluating treatment for LUTS, evidence for the validity of the PGI-S and PGI-I was provided through the associations observed with other symptom and quality of life assessments, but not with peak flow rate severity or changes. The validation of these 2 questionnaires supports their utility as simple tools in research or for clinicians who treat patients with BPH- LUTS.

Symptom severity at V2 as assessed by PGI-S and changes in symptoms as assessed by PGI-I at endpoint were moderately to highly correlated with scores from other BPH patient-reported measures. Further support for the validity of the PGI-S and PGI-I was provided when similar associations were observed across ethnicity and V2 severity (as categorized by IPSS scores). (Tables 3 and 5).

The association with the clinical parameter, peakflow (Qmax), was weak both at V2 and endpoint (Tables 2 and 4). The weak correlation between subjective scores such as PGI-S and PGI-I and Qmax or Qmax change is not surprising. When Qmax was evaluated in the recently updated AUA BPH treatment guidelines 2010, the AUA panel concluded that Qmax correlates poorly with subjective symptoms, PVR and prostate size, and is a weak, patient-oriented outcome in that the patient only marginally experiences flow rate differences. The AUA panel also found that Qmax is not particularly useful from a diagnostic point of view [4]. A high correlation between symptoms scores but a weak correlation with objective parameters (including Qmax) has been reported before in studies within the same BPH disease area as well as in other areas such as female stress incontinence. When Angalakuditi and coauthors validated the BII instrument against IPSS, IPSS-QoL, PVR and Qmax in 12-week placebo-controlled studies, they found a high correlation with patient-reported symptoms and a poor correlation with objective parameters [6]. Yalcin and Bump examined the construct validity of PGI-S and PGI-I against stress incontinence episodes (assessed with weekly diaries), the Incontinence Quality of Life Questionnaire (I-QoL) measures, and leakage during physical exertion (per pad test), and identified weak correlation with the objective pad test in contrast to the higher correlation with the other instruments [9]. It is interesting to note that across different lower urinary tract conditions, objective parameters do not seem to sufficiently capture patient-reported symptoms. These findings confirm the need to include a patient-reported assessment in the evaluation of urological treatment benefit.

As in this study, moderate to high correlations (0.4 to 0.6) have been shown between patient global assessments and symptom measures in other therapeutic areas. It may be expected that a global assessment of symptom severity or change in symptom burden would correlate more highly (e.g., >0.70) with a symptom measure; for example, the association between PGI-S and PGI-I and the IPSS would be stronger than reported if it adequately captured symptom burden. However, there are factors that potentially affect the strength of the association. One factor is the recall period for the measures. The IPSS has a month recall period in which respondents are to recall and purportedly average their symptom severity over that month, whereas the PGI-S asks respondents to report current severity. At endpoint, the IPSS asks the respondent to recall only the past month and this is statistically compared to the respondent’s recall of the month prior to V2 (in which they may have been receiving treatment for BPH-LUTS, erectile dysfuncion, overactive bladder, etc.), while the PGI-I asks the respondent to remember how their symptoms were before they believed they were receiving treatment medication and compare that to how their symptoms are currently. Without asking the participants what timeframe they were considering when completing these instruments, it is difficult to estimate the effect that the difference in recall periods has upon the strength of the associations between the IPSS and the global assessments.

Another factor that may affect the magnitude of the correlation is that the urinary symptoms that the participants are asked to consider are not completely identical for each questionnaire. The IPSS does not include all urinary symptoms that could be affecting a patient with BPH-LUTS. While in the global impression scales implemented in the tadalafil clinical studies and validated here, examples of urinary symptoms were provided based on the symptoms in the IPSS questionnaire [3], in addition to a few other symptoms. In PGI-I and PGI-S, terminal dribble was included as an example of urinary symptoms because it has been identified as a prevalent LUTS in many men [16] and may be associated with BPH. Postmicturition dribble defined as urine leakage almost immediately after finishing urinating and walking away from the toilet was omitted as an example of urinary symptom in the PGI-S and PGI-I, since accidental urinary leakage was already included. Dysuria was also omitted as an example of urinary symptoms since the exclusion criteria in the tadalafil studies typically would eliminate patients with such presentation. Overall, the different urinary symptoms provided as examples in the PGI-S and PGI-I were not meant to be exhaustive of all urinary symptoms or restrictive to BPH, but aimed at providing the patient with examples of common urinary symptoms he could experience. In contrast, the global impression scale tested by Yalcin and Bump did not provide any examples or explanation of the urinary condition tested [9].

Despite the inherent differences between the global assessments and the IPSS, as well as the other patient-reported measures, the relationships observed in this study were in the direction hypothesized and showed the greatest changes in self-reported symptoms, quality of life, and BPH impact for those who perceived themselves as improved (i.e., getting better).

A slightly different patient global assessment of improvement has been included before in a placebo-controlled 1-year study assessing changes to mono-therapy or combination-therapy with an alpha-blocker and/or 5 alpha-reductase-inhibitor in men with BPH-LUTS [19]. The authors concluded that the global assessments of improvement attested to the clinical significance of the difference in other outcome measures [19]. While the construct validity demonstrated in the analysis presented in our paper is applicable to a population of men with BPH-LUTS, the validity of the PGI-S and PGI-I for men with other lower urinary tract symptoms has not been established.

Since the development of the IPSS or BII in the beginning of the nineties, the urological environment and treatment culture has changed from surgery as the cornerstone of BPH treatment to predominantly drug therapy. A simple 1-item global questionnaire may, therefore, seem more applicable today.

## Conclusions

Evidence of the construct validity of the PGI-S and PGI-I for men with BPH-LUTS provided in this study indicates that the 2 instruments could be a valuable and useful tool for clinical studies and practice. While the regulatory evaluation of treatment benefit in clinical trials may require multi-item instruments to fully describe the impact of treatment on various symptoms [20], the PGI-S and PGI-I can provide an overall appraisal of a patients’ condition and is more practical for clinical use by its simplicity in administration and interpretability.

## Competing interests

All the authors are employees of Eli Lilly and Company, the manufacturer of Cialis®, a PDE5-inhibitor, which is approved in USA, EU and other geographies around the world for the treatment of ED, BPH-LUTS (signs and symptoms of BPH) and ED/BPH-LUTS.

## Authors’ contributions

LV was responsible for conception and study design, data interpretation, manuscript writing, critical review of the final manuscript and overall project management; WS and PW were responsible for study design, data analysis, manuscript writing and critical review of the final manuscript; RH was responsible for study design, data interpretation, manuscript writing and critical review of the final manuscript. All authors read and approved the final manuscript.

## Pre-publication history

The pre-publication history for this paper can be accessed here:

http://www.biomedcentral.com/1471-2490/12/30/prepub

## Supplementary Material

Additional file 1 International Prostate Symptom Score (IPSS). Click here for file

Additional file 2 BPH Impact Index (BII). Click here for file
