# Supplementary material for: Construct validation of patient global impression of severity (PGI-S) and improvement (PGI-I) questionnaires in the treatment of men with lower urinary tract symptoms secondary to benign prostatic hyperplasia
Source: BMC Urol. 2012 Nov 7;12:30. doi: 10.1186/1471-2490-12-30 (PMC3503561; doi:10.1186/1471-2490-12-30)
Supplement: Additional file 1 — International Prostate Symptom Score (IPSS). [file 1471-2490-12-30-S1.docx]

# Additional files

Additional file 1 – **International Prostate Symptom Score (IPSS)**

| **In the past month** | **Not at All** | **Less than 1 in 5 Times** | **Less than Half the Time** | **About Half the Time** | **More than Half the Time** | **Almost Always** | **Your Score** |
| --- | --- | --- | --- | --- | --- | --- | --- |
| **1.Incomplete Emptying**  How often have you had the sensation of not emptying your bladder? | 0 | 1 | 2 | 3 | 4 | 5 |  |
| **2.Frequency**  How often have you had to urinate less than every two hours? | 0 | 1 | 2 | 3 | 4 | 5 |  |
| **3.Intermittency**  How often have you found you stopped and started again several times when you urinated? | 0 | 1 | 2 | 3 | 4 | 5 |  |
| **4.Urgency**  How often have you found it difficult to postpone urination? | 0 | 1 | 2 | 3 | 4 | 5 |  |
| **5.Weak Stream**  How often have you had a weak urinary stream? | 0 | 1 | 2 | 3 | 4 | 5 |  |
| **6.Straining**  How often have you had to strain to start urination? | 0 | 1 | 2 | 3 | 4 | 5 |  |
|  | **None** | **1 Time** | **2 Times** | **3 Times** | **4 Times** | **5Times** |  |
| **7. Nocturia**  How many times did you typically get up at night to urinate? | 0 | 1 | 2 | 3 | 4 | 5 |  |

| **Quality of Life due to Urinary Symptoms** | **Delighted** | **Pleased** | **Mostly Satisfied** | **Mixed** | **Mostly Dissatisfied** | **Unhappy** | **Terrible** |
| --- | --- | --- | --- | --- | --- | --- | --- |
| If you were to spend the rest of your life with your urinary condition just the way it is now, how would you feel about that? | 0 | 1 | 2 | 3 | 4 | 5 | 6 |
