# Supplementary material for: Construct validation of patient global impression of severity (PGI-S) and improvement (PGI-I) questionnaires in the treatment of men with lower urinary tract symptoms secondary to benign prostatic hyperplasia
Source: BMC Urol. 2012 Nov 7;12:30. doi: 10.1186/1471-2490-12-30 (PMC3503561; doi:10.1186/1471-2490-12-30)
Supplement: Additional file 2 — BPH Impact Index (BII). [file 1471-2490-12-30-S2.docx]

# Additional files

Additional file 2 – **BPH Impact Index (BII)**

|  | **None** | **Only a little** | **Same** | **A lot** |  |
| --- | --- | --- | --- | --- | --- |
| 1. Over the past month, how much physical discomfort did any urinary problems cause you? | 0 | 1 | 2 | 3 |  |
| 2. Over the past month, how much did you worry about your health because of any urinary problems? | 0 | 1 | 2 | 3 |  |
|  | **Not at all bothersome** | **Bothers me a little** | **Bothers me some** | **Bothers me a lot** |  |
| 3. Overall, how bothersome has any trouble with urination been during the past month? | 0 | 1 | 2 | 3 |  |
|  | **None of the time** | **A little of the time** | **Some of the time** | **Most of the time** | **All of the time** |
| 4. Over the past month, how much of the time has any urinary problem kept you from doing the kinds of things you would usually do? | 0 | 1 | 2 | 3 | 4 |
